# Wellness School Assessment Tool Version 3.0: An Updated Quantitative Measure of Written School Wellness Policies

**DOI:** 10.5888/pcd17.190373

**Published:** 2020-07-02

**Authors:** Marlene B. Schwartz, Elizabeth Piekarz-Porter, Margaret A. Read, Jamie F. Chriqui

**Affiliations:** 1 Rudd Center for Food Policy and Obesity, University of Connecticut, Hartford, Connecticut; 2 Institute for Health Research and Policy, School of Public Health, University of Illinois at Chicago, Chicago, Illinois

## Abstract

Schools play an important role in promoting student wellness. As directed by the Healthy Hunger-Free Kids Act, the US Department of Agriculture updated the requirements for written school wellness policies in 2016. The WellSAT (Wellness School Assessment Tool) is an online tool that provides a quantitative score for wellness policy comprehensiveness and strength. The WellSAT has been updated 3 times over the past decade to remain current with federal law and best practices. In this article, we describe the process of updating to WellSAT 3.0. The steps included: reviewing the language of each item linked to a federal provision; adding and deleting items based on frequencies from the National Wellness Policy Study and the empirical support for specific policies; gathering feedback from a survey of experts (N = 77) about best practices and measure usability; and establishing intercoder reliability in a national sample (N = 50) of policies. We conclude with recommendations and guidance for the use of WellSAT 3.0.

**Summary**

**What is already known on this topic?**

Current federal regulations require most school districts to consider evidence-based strategies to address student wellness and write policies that outline practices related to school food, nutrition, and physical activity.

**What is added by this report?**

Previous versions of a free online tool, the Wellness School Assessment Tool (www.WellSAT.org), have been used over the past decade to provide a quantitative, reliable assessment of the comprehensiveness and strength of these policies for administrators, researchers, and other stakeholders.

**What are the implications for public health practice?**

This article describes how the measure was updated in 2018 to WellSAT 3.0 in response to changes in the federal law, research, and expert feedback.

## Background

Over the past 15 years, the role of schools in promoting wellness has been in the spotlight (1–3). Policies and regulations have been implemented at the state level (4) and federal level, most recently through the Healthy Hunger-Free Kids Act of 2010 (HHFKA) (5–8). In addition, since 2006, local district wellness policies (hereafter School Wellness Policies [SWPs]) have been required in all school districts participating in federal child nutrition programs (9,10).

Originally, SWPs were required to include goals for nutrition education and physical activity to promote student wellness; nutritional guidelines for all foods available on each school campus during the school day; an assurance that reimbursable school meals follow federal law; a plan for measuring policy implementation; and the involvement of parents, students, the school food authority, school board, school administrators, and the public in the development of the policy (9). However, the HHFKA (8) required the US Department of Agriculture (USDA) to update its regulations concerning SWPs, and new regulations were put into effect in 2016 (7). Currently, SWPs must also ensure that school districts review and consider evidence-based strategies to promote student wellness; comply with federal competitive food nutrition standards (ie, Smart Snacks [6]) for all foods sold on campus during the school day; prohibit marketing of all foods and beverages that do not meet Smart Snacks standards; permit all stakeholders to participate in policy development, implementation, review, and updates; report annually to the community about policy content, implementation, and updates; complete triennial assessments on compliance, alignment with model policies, and progress toward goals; and designate one or more officials as responsible for school-level compliance with the policy.

### Original assessment measure for SWPs

To support the empirical study of SWPs, a 96-item quantitative measure was published in 2009 (11). Policy items were identified by reviewing model policies, district self-evaluation tools, state checklists, and state policy classification systems by the National Cancer Institute (12–14). A rubric was developed to assess how completely each item was addressed, and each item was assigned a corresponding score: topic not addressed at all, score = 0; topic mentioned in a general manner or as part of a recommendation, score = 1; topic addressed in a specific manner or as part of a requirement, score = 2. The tool provided 2 summary scores: comprehensiveness, the percentage of items that received a 1 or 2; and strength, the percentage of items that received a 2. This scoring distinguished districts with strong policies that require or prohibit a practice (eg, “Teachers are prohibited from using food as a reward”) while also acknowledging that districts may take an incremental approach to policy change by beginning with aspirational recommendations (eg, “We recommend that teachers use nonfood rewards”).

### Measure evolution

Over time, this measure was adapted to meet needs in the field. First, Chriqui and colleagues expanded it for the largest, ongoing nationwide assessment of SWPs. This endeavor originated in the Bridging the Gap research program and continued in the National Wellness Policy Study (NWPS) (2,15). To date, more than 5,000 districts nationally have been coded to provide reliable data on the content and strength of SWPs over time (2,10).

Second, in 2010 a national advisory group shortened the measure, named it the WellSAT (Wellness School Assessment Tool), and placed it on a free website, www.wellsat.org. WellSAT 1.0 had 50 items and 6 subscales: nutrition education (NE); standards for USDA school meals (SM); nutrition standards for competitive and other foods and beverages (NS); physical education and physical activity (PEPA); wellness promotion and marketing (WPM); and implementation, evaluation, and communication (IEC). After coding, the user receives 1) each item’s score, 2) the comprehensiveness and strength scores for each subscale, and 3) the comprehensiveness and strength scores for the whole policy. Users can code multiple policies and download scores into an Excel file. Since its launch, the website has been accessed more than 100,000 times, and nearly 7,000 registered users have coded more than 9,000 policies from all 50 states and Washington, DC.

In 2014, WellSAT 2.0 was released to reflect the strengthened school meal standards of the HHFKA (5). After the final wellness policy rule requirements for school year 2017–18 were released (6,7,16), the measure and website were updated to version 3.0. This article describes the update and guidance for users of the tool. This study was determined to be exempt from institutional review board approval.

## Updating to WellSAT Version 3.0

To revise the measure, the research team reviewed the language of each item inked to a federal provision; examined item frequencies from the NWPS; reviewed the empirical support for specific policies that were under consideration for inclusion or removal; gathered feedback from a survey of experts about best practices and measure usability; and established intercoder reliability. Table 1 lists the 19 WellSAT 3.0 variables that are based on a federal requirement, and Table 2 provides the final 67 WellSAT 3.0 items, the 12 WellSAT 2.0 items that were removed, and the reasons for the changes. The final measure is available at www.wellsat.org. 

**Table 1:** Federal Wellness Policy Required Elements and Corresponding WellSAT 3.0 Variable

Required Element of School Wellness Policies (7)	WellSAT 3.0 Variable
Specific goals for nutrition promotion and education, physical activity, and other school-based activities that promote student wellness. In developing these goals, local education agencies must review and consider evidence-based strategies and techniques; §210.31 (c)(1)	NE1
Nutrition guidelines for all foods and beverages available on each school campus during the school day that are consistent with federal school meal standards and Smart Snacks in School nutrition standards; §210.31 (c)(3)	SM1, NS1, NS3, NS4, NS5, NS6, NS7
Nutrition standards for all foods and beverages provided, but not sold, to students during the school day; §210.31 (c)(2)	NS9^a^
Policies for food and beverage marketing on school campus during the school day of only those foods and beverages that meet Smart Snacks in School; §210.31 (c)(3)(iii)	WPM7
A description of the manner in which stakeholders (parents, students, teachers, school food authority, teachers of physical education, school health professionals, school board, school administrators, and the public) are provided an opportunity to participate in policy development, implementation, review, and update; §210.31 (c)(5)	IEC2
A requirement that the district inform the public about the content and implementation of the local wellness policy, and make the policy and any updates to the policy available to the public on an annual basis; §210.31 (d)(2)	IEC4
A requirement that at least once every 3 years, the district assess schools’ compliance with the local wellness policy, and make the assessment results available to the public; §210.31 (e)(2)	IEC5, IEC6
A requirement that the district make appropriate updates or modifications to the local wellness policy, based on the triennial assessment; §210.31 (e)(3)	IEC7
Identification of one or more district and/or school officials as wellness policy leadership who are responsible for the implementation and oversight of the wellness policy to ensure each school’s compliance; §210.31 (e)(1)	IEC3
**Healthy, Hunger Free Kids Act of 2010 **(8)	
Schools must make potable water available to children at no charge in the place where lunches are served during the meal service; §203	SM8
**Professional Standards for State and Local School Nutrition Programs Personnel **(17)	
Annual training requirements include 12 h for new and current directors, 10 h for new and current managers, 6 h for new and current staff; §210.30 (e)	SM9
**National School Lunch Act **(18)	
School food authorities must ensure that a child’s eligibility status is not disclosed at any point in the process of providing free or reduced-price meals; §1758(b)(10)	SM3

Abbreviations: IEC, Implementation, Evaluation, and Communication; NE, nutrition education; NS, Nutrition Standards for Competitive and Other Foods and Beverages; SM, Standards for USDA School Meals; WellSAT, Wellness School Assessment Tool; WPM, Wellness Promotion and Marketing.

a This variable assesses elementary school policies; the federal language is general and does not specify school level.

**Table 2:** Final WellSAT 3.0 Items, Removed 2.0 Items, and Reasons for Changes

WellSAT 3.0 Item		Change	Reason for Change
**Nutrition Education (NE)**			
NE1	Federal requirement: includes goals for nutrition education that are designed to promote student wellness	Wording revised from 2.0 NE1 to reflect federal language.	Reflect final rule language
NE2	Nutrition education teaches skills that are behavior focused, interactive, and participatory	Wording revised from 2.0 NE6	Feedback from expert survey
NE3	All elementary school students receive sequential and comprehensive nutrition education	Wording revised from 2.0 NE2 to add “sequential and comprehensive”	Feedback from expert survey
NE4	All middle school students receive sequential and comprehensive nutrition education	Wording revised from 2.0 NE3 to add “sequential and comprehensive”	Feedback from expert survey
NE5	All high school students receive sequential and comprehensive nutrition education	Wording revised from 2.0 NE4 to add “sequential and comprehensive”	Feedback from expert survey
NE6	Nutrition education is integrated into other subjects beyond health education	New item	NWPS found 25% weak and 35% strong policies
NE7	Nutrition education is linked with the school food environment	Wording revised from 2.0 NE5	Feedback from expert survey
NE8	Farm to School item: Nutrition education addresses agriculture and the food system	New item	Feedback from expert survey and Farm to School best practices (19)
**Standards for USDA School Meals (SM)**			
SM1	Federal requirement: assures compliance with USDA nutrition standards for reimbursable school meals.	Wording revised from 2.0 SM2 to add “assures”	Reflect final rule language
SM2	Addresses access to the USDA School Breakfast Program	No change	
SM3	Federal requirement: district takes steps to protect the privacy of students who qualify for free or reduced-priced meals	Wording revised from 2.0 SM4 to note this is a federal requirement	It is a federal requirement
SM4	Addresses how to handle feeding children with unpaid meal balances without stigmatizing them	New item	Reflects current issue in the field (20)
SM5	Specifies how families are provided information about determining eligibility for free/reduced-price meals	No change	
SM6	Specifies strategies to increase participation in school meal programs	No change	
SM7	Addresses the amount of “seat time” students have to eat school meals	Wording revised from 2.0 SM8 to add “seat time”	Feedback from expert survey
SM8	Federal requirement: free drinking water is available during meals	Wording revised from 2.0 SM14 to note this is a federal requirement	Reflect final rule language
SM9	Federal requirement: ensures annual training for food and nutrition services staff in accordance with USDA professional standards	No change	
SM10	Farm to School item: addresses purchasing local foods for the school meal program	New item	Feedback from expert survey and Farm to School best practices
**Nutrition Standards for Competitive and Other Foods and Beverages (NS)**			
NS1	Federal requirement: assures compliance with USDA nutrition standards (commonly referred to as Smart Snacks) for all food and beverages sold to students during the school day	Wording revised from 2.0 NS1 to add “assures”	Reflect final rule language
NS2	USDA Smart Snack standards are easily accessed in the policy	Wording revised from 2.0 NS9 to say “easily accessed” instead of requiring standards are described in full or provided in a link	Feedback from expert survey
NS3	Federal requirement: regulates food and beverages sold a la carte	New item	Venue-specific item to be consistent with NWPS
NS4	Federal requirement: regulates food and beverages sold in vending machines	New item	Venue-specific item to be consistent with NWPS
NS5	Federal requirement: regulates food and beverages sold in school stores	New item	Venue-specific item to be consistent with NWPS
NS6	Addresses fundraising with food to be consumed during the school day	New item	Reflect final rule language
NS7	Exemptions for infrequent school-sponsored fundraisers	New item	Reflect final rule language
NS8	Addresses foods and beverages containing caffeine at the high school level	No change	
NS9	Federal requirement: regulates food and beverages served at class parties and other school celebrations in elementary schools	Wording revised from 2.0 NS4 to combine food and beverages and indicate it is now a federal requirement	NWPS data indicate that this issue is addressed significantly more frequently in elementary schools than middle or high schools. Therefore, this item remains specific to elementary schools. However, the federal rule language is general and does not specify school level.
NS10	Addresses nutrition standards for all foods and beverages served to students after the school day, including before/after care on school grounds, clubs, after-school programming	Wording revised from 2.0 NS3 to define “after the school day” to include clubs and after-school programming	Feedback from expert survey
NS11	Addresses nutrition standards for all foods and beverages sold to students after the school day, including before/after care on school grounds, clubs, after-school programming	Wording revised from 2.0 NS2 to combine food and beverages and include before and aftercare snacks	Feedback from expert survey
NS12	Addresses food not being used as a reward	Moved from WPM subscale	Feedback from expert survey
NS13	Addresses availability of free drinking water throughout the school day	No change	
**Physical Education and Physical Activity (PEPA)**			
PEPA1	CSPAP: There is a written physical education curriculum for grades K-12.	Added that this is a component of CSPAP	Feedback from expert survey to create separate items for all CSPAP components
PEPA2	The written physical education curriculum for each grade is aligned with national and/or state physical education standards	Added “for each grade”	Feedback from expert survey
PEPA3	Physical education promotes a physically active lifestyle	New item	Added because of high frequency (70%) in NWPS and feedback from expert survey
PEPA4	Addresses time per week of physical education for all elementary school students	No change	
PEPA5	Addresses time per week of physical education for all middle school students	No change	
PEPA6	Addresses time per week of physical education for all high school students	No change	
PEPA7	Addresses qualifications for physical education teachers for grades K-12	No change	
PEPA8	Addresses providing physical education training for physical education teachers	No change	
PEPA9	Addresses physical education exemption requirements for all students	No change	
PEPA10	Addresses physical education substitution for all students	No change	
PEPA11	CSPAP: Addresses family and community engagement in physical activity opportunities at all schools	Added that this is a component of CSPAP	Feedback from expert survey to create separate items for all CSPAP components
PEPA12	CSPAP: Addresses before- and after-school physical activity for all students including clubs, intramural, and interscholastic opportunities	Added that this is a component of CSPAP	Feedback from expert survey to create separate items for all CSPAP components
PEPA13	Addresses recess for elementary school students	Wording revision from 2.0 PEPA15 to include only elementary schools	To be consistent with NWPS
PEPA14	CSPAP: Addresses physical activity breaks for all K-12 students	Added that this a component of CSPAP	Feedback from expert survey
PEPA15	Joint or shared-use agreements for physical activity participation at all schools	No change	
PEPA16	Addresses active transport (Safe Routes to School) for all K-12 students who live within walkable/bikeable distance	Wording revised from 2.0 PEPA13 to add “Safe Routes to School”	Feedback from expert survey
**Wellness Promotion and Marketing (WPM)**			
WPM1	Encourages staff to model healthy eating and physical activity behaviors	Wording revised to combine staff modeling of healthy eating and physical activity behaviors	To be consistent with NWPS
WPM2	CSPAP: Addresses strategies to support employee wellness	New item to address employee wellness	To be consistent with NWPS and feedback from expert survey to address employee wellness broadly; also serves as separate item for staff component of CSPAP
WPM3	Addresses using physical activity as a reward	No change	
WPM4	Addresses physical activity not being used as punishment	No change	
WPM5	Addresses physical activity not being withheld as punishment	No change	
WPM6	Specifies marketing to promote healthy food and beverage choices	No change	
WPM7	Federal requirement: restricts marketing on the school campus during the school day to only those foods and beverages that meet Smart Snacks standards	Wording revised to note that this is a federal requirement	Reflect final rule language
WPM8	Specifically addresses marketing on school property (eg, signs, scoreboards, sports equipment)	Wording revised from 2.0 WPM11 for clarity	Feedback from expert survey
WPM9	Specifically addresses marketing of educational materials (eg, curricula, textbooks, or printed or electronic educational materials)	Wording revised from 2.0 WPM12 for clarity	Feedback from expert survey
WPM10	Specifically addresses marketing where food is purchased (eg, exteriors of vending machines, food or beverage cups or containers, food display racks, coolers, trash and recycling containers)	Wording revised from 2.0 WPM13 for clarity	Feedback from expert survey
WPM11	Specifically addresses marketing in school publications and media (eg, advertisements in school publications, school radio stations, in-school television, computer screen savers, school-sponsored internet sites, and announcements on the public announcement [PA] system)	Wording revised from 2.0 WPM14 for clarity	Feedback from expert survey
WPM12	Specifically addresses marketing through fundraisers and corporate incentive programs (eg, fundraising programs that encourage students and their families to sell, purchase, or consume products and corporate incentive programs that provide funds to schools in exchange for proof of purchases of company products such as Box Tops for Education)	Wording revised from 2.0 WPM15 and broadened	Feedback from expert survey
**Implementation, Evaluation, and Communication (IEC)**			
IEC1	Addresses the establishment of an ongoing district wellness committee	No change	
IEC2	Federal requirement: addresses how all relevant stakeholders (parents, students, representatives of the school food authority, teachers of physical education, school health professionals, the school board, school administrator, and the general public) will participate in the development, implementation, and periodic review and update of the local wellness policy	Wording revised to note this is a federal requirement	Reflect final rule language
IEC3	Federal requirement: identifies the officials responsible for the implementation of and compliance with the local wellness policy	Wording revised to note this is a federal requirement	Reflect final rule language
IEC4	Federal requirement: addresses making the wellness policy available to the public annually	Updated from 2.0 IEC5 to reflect federal requirement	Reflect final rule language
IEC5	Federal requirement. addresses the assessment of district implementation of the local wellness policy at least once every 3 years	Updated from 2.0 IEC5 to reflect triennial assessment	Reflect final rule language
IEC6	Triennial assessment results will be made available to the public and will include the extent to which schools under the jurisdiction of the LEA are in compliance with the local school wellness policy the extent to which the LEA’s local school wellness policy compares to model local school wellness policies; and the description of the progress made in attaining the goals of the local school wellness policy.	Updated from 2.0 IEC7 to include the specific components in the federal requirement	Reflect final rule language
IEC7	Federal requirement: addresses a plan for updating the policy based on results of the triennial assessment	Wording revised from 2.0 IEC9 to note this is a federal requirement based on the triennial assessment	Reflect final rule language
IEC8	Addresses the establishment of an ongoing school building–level wellness committee.	New item	Feedback from expert survey and best practice (21)
**Former WellSAT 2.0 Item**			
2.0 SM3	School meals meet standards that are more stringent than those required by USDA	Removed	Unnecessary due to updated USDA standards per HHFKA
2.0 SM7	Addresses students leaving school during lunch periods	Removed	Low frequency (6%) in NWPS data
2.0 SM10	Addresses school meal environment	Removed	Lack of specificity and interpretability
2.0 SM11	Nutrition information for school meals (eg, calories, saturated fat, sodium, sugar) is available to students and parents	Removed	Unnecessary due to updated USDA standards per HHFKA
2.0 SM13	Recess (when offered) is scheduled before lunch in elementary schools	Removed	Lack of empirical support (22,23) and concerns about feasibility from experts
2.0 NS7	Addresses foods and beverages containing non-nutritive sweeteners (High School)	Removed	Low frequency in NWPS data set (4% for food, 10% for beverages) and current scientific guidance (24)
2.0 PEPA6	Addresses teacher:student ratio for physical education classes	Removed	Low frequency (15%) in NWPS data set
2.0 PEPA9	Addresses physical education waiver requirements for K-12 students	Removed	Low frequency (10%) and because this occurs at state, not district level
2.0 PEPA12	District addresses the development of a CSPAP plan at each school	Removed	Removed because CSPAP components are now separated and noted for each relevant item
2.0 PEPA17	Addresses staff involvement in physical activity opportunities at all schools	Removed	Low frequency (18%). Expert feedback to address staff wellness broadly. NWPS variable on staff wellness added instead (3.0 WPM2)
2.0 PEPA19	District provides physical activity training for all teachers	Removed	For consistency with NWPS
2.0 WPM1	Addresses staff not modeling unhealthy eating/drinking behaviors	Removed	For consistency with NWPS Positive staff role modeling is addressed in 3.0 WPM1

Abbreviations: CSPAP, comprehensive school physical activity program; HHFKA, Healthy Hunger-Free Kids Act; LEA, Local Education Agency; NWPS, National Wellness Policy Study; USDA, US Department of Agriculture; WPM, wellness promotion and marketing; WellSAT, Wellness School Assessment Tool.

### Updates related to federal provisions

First, we edited and added items to reflect updated federal language (6,7,16): NE1, SM1, SM8, NS1, NS6, NS7, NS9, WPM7, IEC2, IEC3, IEC4, IEC5, IEC6, IEC7. The item concerning privacy for students receiving free/reduced lunch (SM3) was also updated by noting that it is a federal requirement.

### NWPS data and current evidence

In our second step, we examined frequency data from the 2014–2015 wave of the NWPS. This wave of 496 district policies was collected from a nationally representative sample of school food authorities that completed the director survey of the USDA’s School Nutrition and Meal Cost Study (10). We used frequency data to inform decisions about adding and removing items. Items that rarely appeared in policies, were no longer relevant because of changes in the federal law, or were not considered best practices or evidence-based were considered for removal. Concurrently, we considered adding emerging topics and additional policies assessed in the NWPS.

All removed items are listed in Table 2. “School meals meet standards that are more stringent than those required by USDA” was originally created because meal standards were not in alignment with the Dietary Guidelines for Americans; however, this item is no longer necessary because of updated federal regulations (5). “Students leaving school during lunch periods” reflected concerns that students on open campuses may be more likely to eat fast food, but closed campus provisions appear infrequently in the NWPS. “Addresses school meal environment” was written to capture cafeteria features, including adequate space and a clean, pleasant environment; however, it is not in the NWPS, and the team determined that the lack of specificity made the item difficult to interpret. “Nutrition information for school meals (eg, calories, saturated fat, sodium, sugar) is available to students and parents” was important before the implementation of the updated nutrition standards (5) but is no longer a priority. “Recess (when offered) is scheduled before lunch in elementary schools” was originally included because it was considered a best practice; however, it was removed because of inconclusive evidence (22,23). “Foods and beverages containing nonnutritive sweeteners in high school” was removed because of low frequency in the NWPS and current scientific guidance (24). “Teacher-student ratio for physical education classes” was removed because of low frequency in the NWPS data set. “Physical education waiver requirements for K-12 students” was also removed owing to low frequency and because waiver provisions are typically governed by state law rather than district policies. “Staff involvement in physical activity opportunities at all schools” was removed owing to low frequency in the NWPS and a new, broader item was added to address staff wellness (WPM2). Finally, “District provides physical activity training for all teachers” and “Staff not modeling unhealthy eating/drinking behaviors” were removed to be consistent with NWPS. The item about positive staff role modeling (WPM1) was retained.

NWPS items were reviewed for inclusion in WellSAT 3.0 if they were present in coded policies and supported as best practices. “Nutrition education integrated into subjects beyond health education” (NE6) was added because it increases opportunities for nutrition education and appears frequently NWPS policies. “How to handle feeding children with unpaid meal balances without stigmatizing them” (SM4) was added because of increasing awareness of problematic practices and advocacy efforts to protect children (20). Because all foods sold during the school day must meet Smart Snacks standards, 3 new venue-specific items were added to be consistent with NWPS: à la carte (NS3), vending machines (NS4), and school stores (NS5). “Physical education promotes a physically active lifestyle” (PEPA3) (25) was added because it is present in 70% of NWPS policies, and “The establishment of an ongoing school building–level wellness committee” (IEC8) was added because it has been identified as a best practice (21).

### Expert survey

The next step was to gather feedback about current best practices and measure usability from tool users and experts on school wellness policies. In April 2018, an online survey was emailed to recently active registered WellSAT 2.0 users (n = ~1,000); the Centers for Disease Control and Prevention (CDC) Nutrition and Obesity Policy Research and Evaluation Network School Wellness Workgroup (n = 146); the Voices for Healthy Kids Nutrition and Physical Activity in Schools Coalition, led by Alliance for a Healthier Generation (n = 25); and experts from CDC (n = 2) and Action for Healthy Kids (n = 2). Some people received the survey more than once because of membership in multiple groups.

In the first part of the survey, we provided the revised list of items under consideration for each subscale and asked respondents to rank the importance of each item as low, medium, or high. There was an open response field for comments and suggested wording modifications next to each item. At the end of each subscale section, we asked, “Please comment on the items as a whole with regard to the policies associated with [name of subscale]. Collectively, do the items capture the range of policy considerations? Are there items you recommend adding or deleting?” The second set of questions included the scoring criteria for each item to achieve a 2 (ie, strong policy) and asked respondents to indicate if the criteria should be “weaker,” “stronger,” or if “it’s just right.” Each question also included a field for comments or wording modifications.

The survey was open for 1 month and was completed by 77 expert users. Respondents were from individual school districts (38%); universities (21%); state/local departments of education or health (14%); national advocacy organizations (18%); state/local advocacy organizations (4%); and national government (4%).

Across all items, an average of 8% of respondents rated items as low importance, 25% rated items as medium importance, and 67% rated items as high importance. The only 2 items rated “low importance” by more than 20% of respondents concerned school gardens and Farm to School activities. In the comments, respondents said that school gardens are not feasible for all schools and coders may not understand which activities are part of “Farm to School.” To respond, the school garden item was removed and instead, gardens were included as an example in a new item on nutrition education addressing agriculture and the food system (NE8). A second new item addressed purchasing local foods for the school meal program (SM10). To help coders, there is a watering can icon by these items and links to the National Farm to School Network website to highlight these core Farm to School elements (19).

The experts recognized the importance of comprehensive physical activity but suggested that instead of 1 item referencing CDC’s Comprehensive School Physical Activity Program (CSPAP) by name (26), there should be items for each component. Thus, the 2.0 CSPAP question was removed and a running person icon identifies the component items: physical education (PEPA1); family and community engagement (PEPA11); physical activity before and after school (PEPA12); and physical activity breaks during school (PEPA14) (26). The fifth CSPAP component concerns staff; however, the 2.0 item, “Staff involvement in physical activity opportunities at all schools,” was found in only 18% of NWPS polices. Furthermore, experts recommended folding this into a broader staff wellness item. Therefore, the NWPS item, “Strategies to address employee wellness” (WPM2) replaced it.

The feedback on the “2” scoring criteria indicated that most experts felt the guidance should stay the same. On average across all items, 9% of the experts thought guidance should be “weaker,” 12% felt it should be “stronger,” and 79% said it was “just right.” Additional comments from the expert survey informed wording changes and clarifications (Table 2). Experts suggested clearly distinguishing items required by federal law, so they are marked with a Capitol building icon, the label “federal requirement,” and exact quotes of federal language with the scoring guidance.

### Intercoder reliability

Our final step was to use the NWPS database of policies collected in the 2014–2015 school year (15) to draw a national sample of 72 school district policies that had been recently updated. From this sample, 50 school districts were chosen if their policy had one or more indicators of incorporating recent USDA rules: 1) Smart Snacks appeared within the text of the policy, 2) water availability at lunch appeared within the text, or 3) the policy achieved high comprehensiveness scores.

Per NWPS protocol, we used internet research with telephone or email follow-up to the superintendent’s office to obtain the 2017–2018 school year policy documents for the 50 identified districts. Because 4 districts were nonresponsive, the next 4 districts identified using the selection criteria were substituted. The SWPs included the board-adopted wellness policy and the associated administrative guidelines, rules, and regulations. Written school wellness policies often list references to other related district policies, state, or federal laws within the text of the wellness policy to communicate that these additional regulations are also recognized by the district; therefore, we also included any additional policies that were incorporated by reference (eg, food service policies, state physical and health education standards). All documents were coded by 2 trained analysts using standard NWPS methods (2,4,10).

Two coders independently coded the policies and assessed reliability in groups of 5 SWPs. The percentage agreement on the first 2 batches was 73% and 76%, κ = 0.56 and κ = 0.60, respectively. The primary coder (E.P.) identified problematic items and wrote additional coding guidance. The percentage agreement on the next 2 sets of 5 polices was 82% and 83%, κ = = 0.71 and κ = 0.72, respectively. Remaining confusion centered on nutrition standards and how district language should be applied to each venue, including fundraisers. To clarify the correct application of Smart Snacks*,* new language was added to introduce the nutrition standards section. The final batch of 5 districts achieved 92% agreement, κ = 0.88. Additional examples were added to the coding guidance to clarify lingering issues. The remaining districts were coded using a consensus coding approach.

## WellSAT 3.0 Data and Uses

Table 3 contains descriptive statistics for each item in the full sample of policies (N = 50). All items were written into at least 1 policy in the sample. The mean comprehensiveness score across all categories of the 54 policies indicated that, on average, districts address more than half of the items on the measure, and the mean strength score of 33 means that only one-third of items include strong, definitive language.

**Table 3:** Distribution of WellSAT 3.0 Coding of No, Weak, or Strong Policy Language and Mean Comprehensiveness and Strength Scores^a^

WellSAT 3.0 Variable Category	No. (%) of Districts (N = 50)			Mean Score (SD)
	No Policy = 0	Weak Policy = 1	Strong Policy = 2	
NE: mean comprehensiveness = 74; mean strength = 55				
NE1. Goals for nutrition education^b^	1 (2)	2 (4)	47 (94)	1.92 (0.34)
NE2. Nutrition education teaches behavior-focused skills	7 (14)	3 (6)	40 (80)	1.66 (0.72)
NE3. Elementary school nutrition education	4 (8)	15 (30)	31 (62)	1.54 (0.65)
NE4. Middle school nutrition education	4 (8)	15 (30)	31 (62)	1.54 (0.65)
NE5. High school nutrition education^c^	5 (11)	14 (31)	26 (58)	1.47 (0.69)
NE6. Nutrition education integrated into other subjects	22 (44)	9 (18)	19 (38)	0.94 (0.91)
NE7. Links nutrition education with school food environment	23 (46)	11 (22)	16 (32)	0.86 (0.88)
NE8. Nutrition education addresses agriculture and the food system	36 (72)	9 (18)	5 (10)	0.38 (0.67)
Standards for USDA Child Nutrition Programs and SM: mean comprehensiveness = 53; mean strength = 27				
SM1. USDA school meals^b^	4 (8)	42 (84)	4 (8)	1.00 (0.40)
SM2. USDA School Breakfast Program	11 (22)	18 (36)	21 (42)	1.20 (0.78)
SM3. Protects privacy of students^b^	24 (48)	9 (18)	17 (34)	0.86 (0.90)
SM4. Unpaid meal charges	44 (88)	3 (6)	3 (6)	0.18 (0.52)
SM5. Eligibility information	23 (46)	7 (14)	20 (40)	0.94 (0.93)
SM6. Strategies to increase participation	13 (26)	9 (18)	28 (56)	1.30 (0.86)
SM7. Seat time for lunch	24 (48)	21 (42)	5 (10)	0.62 (0.67)
SM8. Water during meals^b^	26 (52)	1 (2)	23 (46)	0.94 (1.00)
SM9. Food service training^b^	23 (46)	20 (40)	7 (14)	0.68 (0.71)
SM10. Local food procurement	41 (82)	2 (4)	7 (14)	0.32 (0.71)
NS for competitive and other foods and beverages: mean comprehensiveness = 59; mean strength = 37				
NS1. Smart Snacks^b^	1 (2)	9 (18)	40 (80)	1.78 (0.46)
NS2. Link to or full text of Smart Snacks	8 (16)	33 (66)	9 (18)	1.02 (0.59)
NS3. A la carte sales^b^	3 (6)	7 (14)	40 (80)	1.74 (0.56)
NS4. Vending machine sales^b^	2 (4)	7 (14)	41 (82)	1.78 (0.51)
NS5. School store sales^b^	3 (6)	7 (14)	40 (80)	1.74 (0.56)
NS6. Fundraisers^b^	0 (0)	8 (16)	42 (84)	1.84 (0.37)
NS7. Exemptions for infrequent school-sponsored fundraisers	38 (76)	10 (20)	2 (4)	0.28 (0.54)
NS8. Caffeine high school^c^	44 (98)	1 (2)	0 (0)	0.02 (0.15)
NS9. Elementary school class parties^b^	17 (34)	33 (66)	0 (0)	0.66 (0.48)
NS10. Food served before/after school day	44 (88)	3 (6)	3 (6)	0.18 (0.52)
NS11. Food sold after school day	43 (86)	6 (12)	1 (2)	0.16 (0.42)
NS12. Food as reward	23 (46)	20 (40)	7 (14)	0.68 (0.71)
NS13. Water throughout day	36 (72)	0 (0)	14 (28)	0.56 (0.91)
PEPA: mean comprehensiveness = 49; mean strength = 28				
PEPA1. PE curriculum	0 (0)	7 (14)	43 (86)	1.86 (0.35)
PEPA2. National or state PE standards	9 (18)	2 (4)	39 (78)	1.60 (0.78)
PEPA3. Physically active lifestyle	7 (14)	2 (4)	41 (82)	1.68 (0.71)
PEPA4. PE time elementary school	31 (62)	16 (32)	3 (6)	0.44 (0.61)
PEPA5. PE time middle school	33 (66)	16 (32)	1 (2)	0.36 (0.53)
PEPA6. PE time high school^c^	40 (89)	4 (9)	1 (2)	0.13 (0.40)
PEPA7. Qualifications for PE teachers	25 (50)	4 (8)	21 (42)	0.92 (0.97)
PEPA8. PE teacher training	32 (64)	5 (10)	13 (26)	0.62 (0.88)
PEPA9. PE exemption	48 (96)	2 (4)	0 (0)	0.04 (0.20)
PEPA10. PE substitution	47 (94)	1 (2)	2 (4)	0.10 (0.42)
PEPA11. Family engagement in PA	22 (44)	24 (48)	4 (8)	0.64 (0.63)
PEPA12. Before/after school PA opportunities	11 (22)	18 (36)	21 (42)	1.20 (0.78)
PEPA13. Recess	19 (38)	25 (50)	6 (12)	0.74 (0.66)
PEPA14. PA breaks	14 (28)	30 (60)	6 (12)	0.84 (0.62)
PEPA15. Joint use	33 (66)	6 (12)	11 (22)	0.56 (0.84)
PEPA16. Safe Routes to School	33 (66)	8 (16)	9 (18)	0.52 (0.79)
WPM: mean comprehensiveness = 32; mean strength = 20				
WPM1. Staff role model	21 (42)	20 (40)	9 (18)	0.76 (0.74)
WPM2. Employee wellness	27 (54)	6 (12)	17 (34)	0.80 (0.93)
WPM3. PA as reward	48 (96)	0 (0)	2 (4)	0.08 (0.40)
WPM4. PA as punishment	28 (56)	8 (16)	14 (28)	0.72 (0.88)
WPM5. PA withheld as punishment	29 (58)	7 (14)	14 (28)	0.70 (0.89)
WPM6. Healthy marketing	22 (44)	4 (8)	24 (48)	1.04 (0.97)
WPM7. Restricted marketing^b^	23 (46)	7 (14)	20 (40)	0.94 (0.93)
WPM8. Marketing on signs	42 (84)	4 (8)	4 (8)	0.24 (0.59)
WPM9. Marketing in education materials	42 (84)	4 (8)	4 (8)	0.24 (0.59)
WPM10. Marketing on vending machines	42 (84)	4 (8)	4 (8)	0.24 (0.59)
WPM11. Marketing in school publications/radio channel 1	42 (84)	4 (8)	4 (8)	0.24 (0.59)
WPM12. Marketing through fundraisers	44 (88)	3 (6)	3 (6)	0.18 (0.52)
IEC: mean comprehensiveness = 70; mean strength = 47				
IEC1. District committee	8 (16)	6 (12)	36 (72)	1.56 (0.76)
IEC2. Stakeholders^b^	6 (12)	24 (48)	20 (40)	1.28 (0.67)
IEC3. Identifies person responsible^b^	9 (18)	7 (14)	34 (68)	1.50 (0.79)
IEC4. Wellness policy available^b^	23 (46)	6 (12)	21 (42)	0.96 (0.95)
IEC5. Triennial assessment^b^	6 (12)	9 (18)	35 (70)	1.58 (0.70)
IEC6. Triennial assessment made available^b^	17 (34)	15 (30)	18 (36)	1.02 (0.84)
IEC7. Update^b^	8 (16)	24 (48)	18 (36)	1.20 (0.70)
IEC8. School committee	44 (88)	1 (2)	5 (10)	0.22 (0.62)

Abbreviations: IEC, Implementation, Evaluation, and Communication; NE, Nutrition Education; SM, Standards for USDA School Meals; NS, Nutrition Standards for Competitive and Other Foods and Beverages; PA, physical activity; PE, physical education; PEPA, Physical Education and Physical Activity; SD, standard deviation; USDA, US Department of Agriculture; WellSAT, Wellness School Assessment Tool; WPM, Wellness Promotion and Marketing.

a Mean overall comprehensiveness score = 54; mean overall strength score = 33.

b This item is reflected in the Federal Rule.

c Fewer than 50 districts were scored for high school items because some districts in the sample did not contain any high schools.

WellSAT 3.0 has multiple uses, including self-evaluation by district wellness committees to comply with the requirement to compare their own policy with model policies and best practices (7). Similarly, state agencies can use it to complement their triennial reviews of SWPs, as required by the USDA (16). State agencies may also find WellSAT useful in providing technical assistance to school districts and tracking environmental policy changes. For example, New York State has used WellSAT to support a cohort of school districts (27), and evaluators in Arizona have used it as one of their SNAP-Ed policy strategies (28).

Although the summary scores are between 0 and 100, they should not be translated into letter grades. The coding reflects the strongest versions of policies, setting the bar to avoid a ceiling effect and help districts strive for excellence. To interpret scores, a stepped interpretation strategy is recommended. First, examine only the items that are noted as federal requirements, and ensure that they are present in the written policy. Next, evaluate each subscale comprehensiveness and strength score. If comprehensiveness is much higher than strength, consider using stronger, more specific language to clarify the expectation of the policy. If comprehensiveness scores are low, pick a subscale consistent with district priorities and consider adding new policies.

### Comparing scores over time

The WellSAT has changed substantially since its inception in 2006. The first version emphasized going beyond federal requirements in multiple domains, including setting strong nutrition standards for school meals and foods and beverages sold outside of meals. The HHFKA now requires many of these practices, so WellSAT 3.0 items assess compliance with federal regulations. However, this measure also promotes further progress, such as extending strong nutrition standards to food sold or served after the school day.

### Limitations

The primary benefit of updating this measure is to reflect changes in federal law and current evidence-based and expert-recommended best practices. However, districts that used WellSAT 2.0 will not be able to track improvements over time. The WellSAT 2.0 tool is available on the website for those districts. Furthermore, the 50 test policies selected were chosen because they were comprehensive, so scores should not be interpreted as nationally representative. However, the results were consistent with the nationally representative NWPS finding that policies address many topics in an aspirational, nonbinding manner (10). Finally, some of the strong scores are due to state laws, not language specific to the SWP (10).

In conclusion, schools are a critical setting for health promotion, and strong SWPs are a key strategy to ensure consistent implementation of best practices. National surveillance is needed, particularly during school year 2020–2021 after the triennial assessments are required. Future research should evaluate the impact of the HHFKA wellness policies and regulations on local policies, practices, and ultimately student and staff health outcomes.
